# The Association of Iatrogenic Withdrawal With Opioid and Benzodiazepine Weaning in Children With Bronchiolitis: A Single-Center, Retrospective Cohort Study, 2012–2022

**DOI:** 10.1097/CCE.0000000000001391

**Published:** 2026-03-13

**Authors:** Alice Shanklin, Eduardo A. Trujillo Rivera, Murray M. Pollack, Anita K. Patel

**Affiliations:** 1 Division of Pediatric Critical Care Medicine, Children’s National Hospital, Washington, DC.; 2 Division of Pediatric Critical Care Medicine, Cohen Children’s Medical Center, Queens, NY.; 3 Research Division of Biostatistics and Study Methodology, Department of Pediatrics, George Washington University, Washington DC.

**Keywords:** alpha-2 agonists (dexmedetomidine, clonidine), critical bronchiolitis, iatrogenic withdrawal

## Abstract

**OBJECTIVES:**

This study aimed to determine the decrease in opioid and benzodiazepine doses associated with the development of withdrawal in children.

**SETTING:**

Electronic health record data.

**INTERVENTION:**

None.

**PATIENTS:**

Four hundred and seven children who received invasive mechanical ventilation (IMV) between January 1, 2012 and January 1, 2022 with Withdrawal Assessment Tool-1 (WAT-1) scores during their IV opioid wean were included.

**MEASUREMENTS AND MAIN RESULTS:**

The primary outcome was development of withdrawal, defined as a WAT-1 score greater than or equal to 3, during the IV opioid weaning phase. Descriptive data included age, weight, insurance, race, ethnicity, language, comorbidities, length of stay, and IMV duration. WAT-1 scores were recorded, and the cumulative IV and enteral opioids, benzodiazepines, and A2As were calculated. Recent changes in IV opioid and benzodiazepine doses were assessed in the 16 hours before each WAT-1 score divided into 4-hour periods. Doses were compared across 4-hour periods, and a multivariable mixed-effects model was used to assess their association with withdrawal. The presence of greater than or equal to 2 pediatric complex chronic condition classification system version 2 (CCC-V2) diagnosis categories (odds ratio [OR] 3.35; 95% CI, 2.61–5.15) was associated with withdrawal. Heart disease, prematurity, and IV opioid class switching did not qualify for model inclusion. Cumulative IV opioid dose and a decrease in opioid exposure between 16–12 and 4–0 hours before the WAT-1 score were associated with withdrawal (OR 1.02; 95% CI, 1.01–1.03; OR 1.92; 95% CI, 1.38–3.16). Morphine was associated with increased odds of withdrawal compared with fentanyl and hydromorphone (OR 1.48; 95% CI, 1.37–1.81). The model did not establish an association between cumulative IV benzodiazepine exposure or IV benzodiazepine dose decrease and withdrawal.

**CONCLUSIONS:**

Patients with a higher cumulative IV opioid dose, an IV opioid wean in the preceding 8–12 hours, and greater than or equal to 2 CCC-V2 categories had the highest odds of withdrawal in this single-center study. Morphine was associated with increased odds of withdrawal as well.

**KEY POINTS**

**Question**: What decrease in opioid and benzodiazepine dose is associated with withdrawal in children intubated for bronchiolitis?

**Findings:** This retrospective, single-center study analyzed the unique medication profiles associated with 18,646 Withdrawal Assessment Tool-1 scores in 407 patients. Greater cumulative IV opioid exposure and a greater decrease in opioid exposure in the preceding 8–12 hours were associated with increased odds of withdrawal. With other factors held constant, a 0.4 mg/kg decrease in the 4-hour IV morphine equivalent dose was associated with 34% increased odds of withdrawal 8–12 hours later.

**Meaning**: Cumulative drug exposure and the IV opioid dose decrease in the preceding 8–12 hours can be used to stratify withdrawal risk.

**RESEARCH IN CONTEXT**

- Withdrawal is a common side effect of opioid and benzodiazepine administration.
- Personalized medication histories were assessed for their association with withdrawal, identified via the Withdrawal Assessment Tool-1 score, in a mixed-effects model.
- A large cohort of children with bronchiolitis who received invasive mechanical ventilation was used to limit heterogeneity of age and diagnosis.

**AT THE BEDSIDE**

- A higher cumulative IV opioid exposure, a greater decrease in opioid exposure from hours 16–12 to 4–0, and the presence of greater than or equal to 2 complex chronic conditions were associated with increased odds of withdrawal.
- Increased cumulative methadone exposure was associated with decreased odds of withdrawal.
- Morphine was associated with higher odds of withdrawal than fentanyl and hydromorphone.

Opioids and benzodiazepines ameliorate suffering, promote comfort, and avoid the adverse physiologic consequences of agitation and pain, particularly in PICU patients (1, 2). Withdrawal is a syndrome induced when reducing or discontinuing the dosage of these medications, especially following cessation of mechanical ventilation (3). It is associated with the cumulative drug dose, the duration of use, the specific drugs being used, and the individual patient’s drug tolerance and metabolism (4). Withdrawal consists of three categories of dysfunction: autonomic dysregulation, overstimulation of the CNS, and gastrointestinal dysfunction. The clinical phenotype includes fever, tachycardia, tachypnea, hypertension, excitability/agitation, irritability, seizures, anxiety, diarrhea, and vomiting (4–6). In PICU patients, opioid and benzodiazepine withdrawal is detected and documented with the Withdrawal Assessment Tool-1 (WAT-1), a scoring instrument of signs and symptoms administered by bedside nurses (4, 7). A separate score exists for A2A withdrawal (WAT-A2A), but it has not been disseminated into clinical practice (8).

Preventing or ameliorating withdrawal episodes could be enhanced by a better understanding of risk at the patient level (3, 9). Most evidence for withdrawal risk stratification comes from small, single-center studies with significant heterogeneity inpatient age, comorbidities, and drug metabolism (5, 6, 10–16). This study investigates, at a granular, patient level, the risk factors associated with withdrawal in a cohort of children with bronchiolitis who received mechanical ventilation, focusing on the specific medications received, cumulative dose, and the changes in doses associated with withdrawal.

## METHODS

The patient sample and all clinical data were obtained from the electronic health record of Children’s National Hospital, a quaternary care center, from January 1, 2012, to January 1, 2022. Data were extracted from HealtheIntent, the institutional data warehouse. The study follows the ethical standards of the Children’s National Hospital Institutional Review Board (protocol number Pro00016929, “Sedation and withdrawal in bronchiolitis,” December 12, 2021, with a waiver of informed consent), and the 1975 Helsinki Declaration. The RECORD checklist was used to create this article (17).

**Figure 1.**
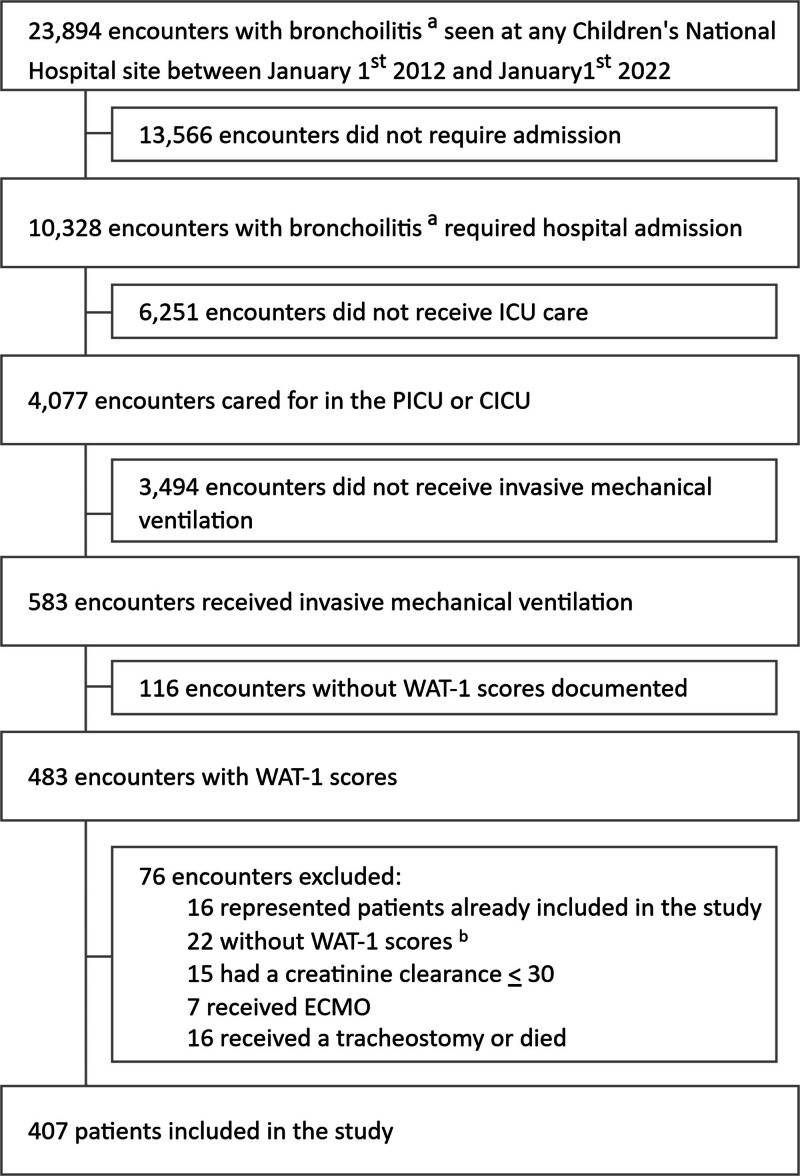
Patient selection. **A**, Bronchiolitis was identified by the presence of *International Classification of Diseases-9* (ICD-9) code 466.1 or ICD-10 code J21 in a patient younger than 24 months (eTable 1, https://links.lww.com/CCX/B612). **B**, Patients without Withdrawal Assessment Tool-1 (WAT-1) scores were documented in the study period. ECMO = extracorporeal membrane oxygenation.

Patients were included if they were under 24 months of age, had bronchiolitis, received invasive mechanical ventilation (IMV), and had WAT-1 score documentation during their IV opioid wean (**Fig. 1**). Bronchiolitis was identified by the presence of *International Classification of Diseases-9* (ICD) code 466.1 or ICD-10 code J21 (**eTable 1**, https://links.lww.com/CCX/B612) (18–21). The IV opioid wean was identified with the first 10% decrease in hourly opioid administration sustained for 24 hours and concluded 24 hours after the opioid infusion was discontinued (22, 23). If a patient had multiple weaning efforts, only the first was included. Patients were excluded if they had evidence of renal dysfunction (estimated creatinine clearance < 30). Patients were also excluded if they were NICU patients, had a tracheostomy, received extracorporeal membrane oxygenation, or died during the hospital admission (Fig. 1) (23).

### Identification of Withdrawal

The primary outcome was the development of withdrawal, defined as a WAT-1 score greater than or equal to 3 (7, 24, 25). Scores were performed routinely every 4–12 hours on patients while weaning opioids or benzodiazepines, with additional scoring performed as clinically indicated (7, 13). Motor Activity Assessment Scale (MAAS) scores were used to assess the level of sedation; WAT-1 scores were excluded if the patient was deeply sedated, defined as a MAAS score less than 2 (26).

### Study Variables

Descriptive data included patient age, insurance, race, ethnicity, language, hospital and ICU length of stay, duration of mechanical ventilation, highest mean airway pressure, and discharge location (**Table 1**; and **Appendix A**, https://links.lww.com/CCX/B612) (30). Comorbidities included heart disease, trisomy 21, and prematurity, defined by ICD-9 or ICD-10 codes (Appendix A, https://links.lww.com/CCX/B612) and greater than or equal to 2 complex chronic conditions, defined by the pediatric complex chronic condition classification system version 2 (CCC V2) diagnosis categories (31). Summative pharmacologic data for each patient included the first IV opioid administered, IV opioid class switching, and the cumulative IV and enteral A2A doses given during the hospitalization, in milligrams per kilogram (**Table 2**). Medication dose calculations included continuous infusions, scheduled doses, and as-needed doses. IV opioid doses were converted to morphine equivalents using a conversion factor of 0.01:1 for fentanyl and 0.15:1 for hydromorphone (28, 29). IV opioid class switching was defined as the administration of a new opioid for at least 6 hours (32). Enteral medications are reported separately to reflect the institutional sedation protocols used during the study period. This protocol recommended enteral methadone or lorazepam after 5–7 days of continuous opioid or benzodiazepine infusion, and enteral clonidine in patients unable to tolerate their A2A infusion wean.

**TABLE 1.** Patient Demographics

Patient Demographics	All Patients, *n* = 407	No Withdrawal^a^, *n* = 64	Withdrawal^a^, *n* = 343
Male, *n* (%)	238 (58.5%)	39 (60.9%)	199 (58.0%)
Age, yr, median (IQR)	0.5 (0.3, 0.9)	0.6 (0.2, 0.9)	0.5 (0.3, 0.9)
Weight, kg, median (IQR)	7.3 (5.6, 9.0)	7.6 (5.5, 9.4)	7.3 (5.6, 9.0)
Insurance, *n* (%)			
Public	286 (70.3%)	44 (68.8%)	242 (70.6%)
Private	110 (27.0%)	18 (28.1%)	92 (26.8%)
Other	11 (2.7%)	2 (3.1%)	9 (2.6%)
Race, *n* (%)			
Black or African American	158 (38.8%)	26 (40.6%)	132 (38.5%)
Caucasian	79 (19.4%)	13 (20.3%)	66 (19.2%)
Asian	9 (2.2%)	3 (4.7%)	6 (1.8%)
Other or not reported	161 (39.6%)	22 (34.4%)	139 (40.5%)
Ethnicity, *n* (%)			
Hispanic/Latino	133 (32.7%)	20 (31.3%)	113 (32.9%)
Not Hispanic/Latino	274 (67.3%)	44 (68.75%)	230 (67.06%)
Language, *n* (%)			
English	274 (67.3%)	44 (68.8%)	230 (67.1%)
Spanish	118 (29.0%)	17 (26.6%)	101 (29.5%)
Other or unknown	15 (3.7%)	3 (4.7%)	12 (3.5%)
Significant comorbidities, *n* (%)			
None	328 (80.6%)	51 (79.7%)	277 (80.8%)
≥ 2 Complex chronic conditions classification system version 2 categories (27)	16 (3.9%)	3 (4.7%)	13 (3.8%)
Cardiac Disease	51 (12.5%)	10 (15.6%)	41 (12.0%)
Prematurity	18 (4.4%)	1 (1.6%)	17 (5.0%)
Trisomy 21	11 (2.7%)	2 (3.1%)	9 (2.6%)
Hospital encounter			
ICU days, median (IQR)	11.4 (8.3, 16.5)	8.7 (6.4, 11.2)	11.9 (8.9, 17.7)
Hospital days, median (IQR)	15.7 (10.8, 22.1)	11.7 (8.6, 16.6)	16.9 (11.8, 23.1)
Discharged to home, *n* (%)	368 (90.4%)	58 (90.6%)	310 (90.4%)
Invasive mechanical ventilation			
Days, median (IQR)	7.3 (4.9, 10.3)	6.2 (3.9, 8.4)	7.5 (5.1, 10.9)
Highest mean airway pressure, cm H_2_O, median (IQR)	18 (16, 20)	17 (16, 19)	18 (16, 20)
≥ 1 Intubation, *n* (%)	42 (10.3%)	3 (4.7%)	39 (11.3%)

IQR = interquartile range.

a Withdrawal is defined as the presence of a Withdrawal Assessment Tool-1 score ≥ 3 during the study time interval.

Continuous variables are presented as median (IQR) and categorical variables as *n* (%).

**TABLE 2.** Opioid and Sedative Medication Exposure

Variable	All Patients, *n* = 407
First IV opioid, *n* (%)	
Morphine	193 (47.4%)
Fentanyl	182 (44.7%)
Hydromorphone	23 (5.7%)
Multiple IV opioids in the first 6 hours	9 (2.2%)
Opioid exposure	
Total IV dose (mg/kg)^a^, median (IQR)	31.8 (19.2, 50.7)
Infusion days, median (IQR)	7.9 (4.9, 10.8)
Average infusion rate^b^, median (IQR)	0.15 (0.11, 0.20)
IV opioid switched, *n* (%)	51 (12.5%)
Enteral opioid (methadone), *n* (%)	283 (69.7%)
Total methadone dose (mg/kg), median (IQR)	3.3 (1.8, 5.7)
Benzodiazepine exposure	
IV midazolam given *n* (%)	386 (94.8%)
Total IV dose (mg/kg), median (IQR)	13.1 (4.4, 28.1)
Infusion days, median (IQR)	4.9 (1.7, 8.8)
Average infusion rate^b^, median (IQR)	0.15 (0.11, 0.25)
Enteral medication (lorazepam), *n* (%)	237 (58.4%)
Total lorazepam dose (mg/kg), median (IQR)	3.0 (1.1, 5.5)
Alpha-2 agonist exposure	
IV dexmedetomidine given, *n* (%)	365 (89.7%)
Total IV dose (µg/kg), median (IQR)	123.3 (60.4, 229.8)
Infusion days, median (IQR)	6.3 (3.3, 10.0)
Average infusion rate^b^, median (IQR)	0.90 (0.63, 1.14)
Enteral medication (clonidine), *n* (%)	79 (19.6%)
Total clonidine dose (µg/kg), median (IQR)	57.0 (31.0, 121.1)
Discharge medications, *n* (%)	
Opioids or sedatives are prescribed	194 (47.7%)
≥ 5 medication days after discharge	128 (31.5%)

IQR = interquartile range.

a IV opioid doses were converted to morphine equivalents using a conversion factor of 0.01:1 for fentanyl and 0.15:1 for hydromorphone (28, 29).

b Average infusion rate, in mg/kg/hr of morphine equivalents, mg/kg/hr of midazolam, and µg/kg/hr of dexmedetomidine.

Cumulative and recent IV and enteral opioid and benzodiazepine doses were calculated for each WAT-1 score, along with the cumulative IV and enteral A2A doses. Cumulative medication dose was measured from the first dose administered in the ICU until the time of WAT-1 documentation. Recent medication doses were assessed in 4-hour periods for the 16 hours before each WAT-1 score (hours 16–12, hours 12–8, hours 8–4, and hours 4–0). The total dose per kg of each IV medication was calculated every 4 hours. Differences in IV medication exposure across the 4-hour periods were used to identify changes in recent exposure (i.e., the difference in medication dose per kg between hours 16–12 and 12–8, or 16–12 and 4–0; **eFig. 1**, https://links.lww.com/CCX/B612).

### Statistical Analysis

Summative statistics for descriptive and pharmacologic variables included counts, percentages, and medians (interquartile ranges [IQRs]).

A multivariable mixed-effects model with time-series data and random effects per patient was used to assess the relationship between variables and withdrawal, and to account for multiple observations within each patient’s IV opioid wean. The multivariable mixed-effects model was created via a non-automated stepwise backward regression with a significance level of *p* value of less than 0.05 for model inclusion. We retained one of the six possible differences in prior IV opioid and benzodiazepine exposure in the final model (i.e., the difference in medication dose between hours 16–12 and 12–8, or hours 16–12 and 4–0) regardless of covariance or significance. The cumulative exposure of each drug class was also retained a priori. The 95% CIs for the model coefficients, odds ratios, and performance metrics for evaluation of model fit were calculated using a nonparametric bootstrap with 9999 samples. Sensitivity thresholds of 0.8 and 0.9 were used to calculate key measures of model fit.

The multivariable mixed-effects model was used to graph the odds of developing withdrawal across a range of IV opioid and benzodiazepine dose decreases between hours 16–12 and hours 4–0. Statistical analyses were completed using R, Version 4.3.2 (R Foundation for Statistical Computing, Vienna, Austria).

## RESULTS

### Study Population

Four hundred and seven patients were included in the study (consort diagram, Fig. 1). Descriptive information is shown in Table 1, and opioid, benzodiazepine, and A2A data are shown in Table 2. Over one-half of the patients (58.5%) were male, and the median age was 0.5 years (IQR 0.2–0.9). Of the cohort, 70.3% had public insurance, 67.3% used English as their primary language, and 38.8% were Black or African American. Most patients did not have a comorbidity (80.6%). Patients received a median of 7.3 days (IQR 4.9–10.3) of IMV, 7.9 days (IQR 4.9–10.8) of IV opioids, 4.9 days (IQR 1.7–8.8) of IV benzodiazepines, and 6.3 days of IV A2As (IQR 3.3–10.0) (Table 2). Morphine was the most common IV opioid (47.4%), followed by fentanyl (44.7%) and hydromorphone (5.7%). A total of 12.5% had their IV opioid class switched during the study period. Over one-half of patients (52.4%) were discharged from the hospital on an enteral opioid, benzodiazepine, or A2A wean.

The mixed-effects model predicting withdrawal included 18,646 WAT-1 scores (**Table 3**). The model’s calibration plot (**eFig. 2**, https://links.lww.com/CCX/B612) and fit descriptors (**eTable 2,** https://links.lww.com/CCX/B612) are available in **Appendix B** (https://links.lww.com/CCX/B612). Of the total WAT-1 scores, 17% (3,167) were greater than or equal to 3, and 83% (15,479) were less than 3. The presence of greater than or equal to 2 complex chronic conditions (31) (OR 4.21; 95% CI, 3.36–6.88) and Trisomy 21 (OR 0.28; 95% CI, 0.13–0.36) qualified for model inclusion, but heart disease, prematurity, and the presence of IV opioid class switching did not. A higher cumulative IV opioid exposure and a greater decrease in opioid exposure from hours 16–12 to 4–0 were associated with increased odds of withdrawal. Higher IV opioid exposure from hours 16–12 and higher cumulative enteric methadone doses were associated with decreased odds of withdrawal. Benzodiazepine exposure showed a different pattern. Increased exposure from hours 16 to 12 was associated with decreased odds of withdrawal, but cumulative exposure and dose decreases were not significant. Morphine, as the first opioid administered, was associated with increased odds of developing withdrawal when compared with any other opioid (OR 1.47; 95% CI, 1.35–1.79). For enteral medications, higher cumulative methadone and clonidine doses were both associated with decreased odds of developing withdrawal (methadone: OR 0.74; 95% CI, 0.63–0.82; clonidine: OR 0.48; 95% CI, 0.30–0.63). Cumulative lorazepam dose was not significant (OR 1.05; 95% CI, 0.92–1.21).

**TABLE 3.** The Odds Ratio of Each Coefficient in the Generalized Linear Mixed-Effects Model

Variable	OR	95% CI
Opioid exposure, mg/kg		
Cumulative IV medication (morphine equivalents) dose^a^	1.02	1.01–1.03
IV morphine equivalent dose decreased from hours 16–12 to 4–0	1.92	1.41–3.21
IV morphine equivalents: hours 16–12	0.37	0.20–0.46
Cumulative enteral medication (methadone) dose	0.74	0.63–0.82
Morphine as the first IV opioid used^b^	1.47	1.35–1.79
Benzodiazepine exposure, mg/kg		
Cumulative IV medication (midazolam) dose	1.00	1.00–1.01
IV midazolam dose decreased from hours 16–12 to 4–0	1.02	0.57–1.77
IV midazolam dose: hours 16–12	0.53	0.29–0.86
Cumulative enteral medication (lorazepam) dose	1.05	0.92–1.21
Alpha-2 agonist exposure, µg/kg dexmedetomidine, mg/kg clonidine		
Cumulative IV medication (dexmedetomidine) dose	1.00	1.00–1.01
Cumulative enteral medication (clonidine) dose	0.48	0.30–0.63
Comorbidities		
≥ 2 Complex chronic conditions classification system version 2 categories	4.21	3.36–6.88
Trisomy 21	0.28	0.13–0.36

OR = odds ratio.

a All IV fentanyl and hydromorphone doses were converted to morphine equivalents.

b Morphine as the first IV opioid used, compared with fentanyl.

**Figure 2.**
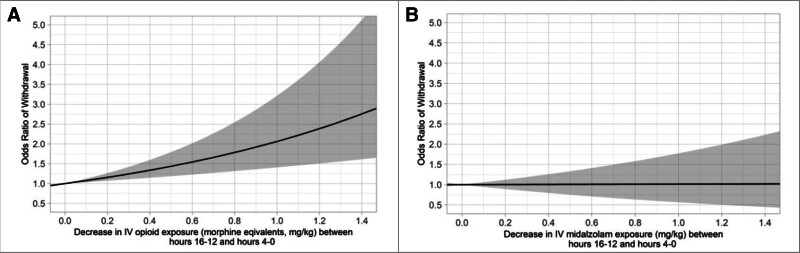
IV opioid and benzodiazepine dose decreases and withdrawal. The odds ratio of developing withdrawal for a variety of decreases in IV opioid dose (**A**) and decreases in IV benzodiazepine (midazolam) dose (**B**). All other model variables are held constant. The 95% CI of the odds ratio is shown in *gray*.

After controlling for the variables in the mixed-effects model, there was a small but significant positive association between higher cumulative IV opioid dose and the odds of developing withdrawal. For example, a patient who received 20.02 mg/kg of IV morphine equivalents has 50% higher odds of withdrawal than a patient who did not have this cumulative opioid dose, with all other factors held constant (OR 1.50; 95% CI, 1.32–1.71). Withdrawal was associated with a decrease in IV opioid dose with all other factors held constant (**Fig. 2**). For example, a 0.4 mg/kg decrease in the 4-hour IV opioid dose between hours 16–12 and hours 4–0 was associated with 34% increased odds of developing withdrawal (**Fig. 2*A***: OR 1.34; 95% CI, 1.15–1.60). The model did not establish an association between the IV benzodiazepine dose decrease between hours 16–12 and hours 4–0 and withdrawal, regardless of the magnitude of the dose decrease (**Fig. 2*B***).

## DISCUSSION

Withdrawal was associated with the presence of greater than or equal to 2 complex chronic conditions, Trisomy 21, a higher cumulative IV opioid dose, and using morphine as the first IV opioid in 407 children mechanically ventilated for bronchiolitis at a single center (31). A greater decrease in IV opioid dose was associated with increased odds of developing withdrawal 8–12 hours in the future in the multivariable mixed-effects model. In contrast, the cumulative IV benzodiazepine dose and recent decreases in IV benzodiazepine dose were not associated with withdrawal. Lastly, increased cumulative methadone dose was associated with decreased odds of withdrawal.

This study confirmed the association between cumulative IV opioid dose and the development of withdrawal. Pediatric intensivists expect a clinically significant withdrawal risk after 5 days of continuous opioid infusion, although robust contemporary data are not available (9, 24). The multivariable model shows that a cumulative dose of 20.02 mg/kg of IV morphine equivalents is associated with 50% increased odds of withdrawal. This suggests that in children with bronchiolitis who require mechanical ventilation in the PICU, the odds of developing withdrawal are increased by 50% after 5.7 days of receiving an average IV opioid infusion rate of 0.15 mg/kg (Table 2) when all other factors are held constant. The multivariable model also allowed for an estimation of the dose effect of IV opioid weans. When all other variables are held constant, a 0.4 mg/kg decrease in the sum of IV opioid exposure over 4 hours between hours 16–12 and 4–0 was associated with 30% increased odds of developing withdrawal (Fig. 2*A*). This 4-hour dose decrease is equivalent to a 0.1 mg/kg decrease in the hourly infusion rate.

In this study, morphine was associated with increased development of withdrawal when compared with other opioids in the multivariable model, and 89% of patients who did not receive morphine received fentanyl. Fentanyl has a shorter time to effect, a shorter duration, and different intracellular signaling pathways than morphine, which leads to increased tolerance, increased tachyphylaxis, and a higher risk of physiologic dependence (3, 27, 33). This finding was therefore unexpected and could be a result of fentanyl’s context-sensitive half-life. Fentanyl is more lipophilic than morphine, resulting in an extended half-life after prolonged exposure (34). Patients receiving fentanyl in our population potentially tolerated a larger decrease in IV opioid dose than patients receiving morphine due to these lipophilic properties.

The model results also showed that Trisomy 21 is protective against withdrawal. Children with Trisomy 21 have differences in forebrain development, GABAergic neuron development, and muscle tone (35). Although this does not result in a clinical difference in pain transmission or analgesia requirement, children with Trisomy 21 exhibit a latent and potentially blunted sensorimotor response to painful stimuli (36, 37). These differences might affect the “response to progressive stimulus,” used in the WAT-1 score. More research is needed to determine if these findings reflect a true decrease in withdrawal or if a more sensitive screening tool is necessary in this population.

### Study Limitations

This study has several important limitations. Patients who were not screened for withdrawal were excluded from the study, which made our population sicker than a typical cohort of children with bronchiolitis who require IMV. In a retrospective, single-center study, distinguishing pharmacologically important results from temporal trends in clinical practice is difficult; however, the mixed-effects model corrects for many important factors. In addition, several variables retained a priori in the multivariable model because of their clinical significance showed covariance, making it difficult to distinguish their independent effect. For example, the cumulative IV benzodiazepine dose exhibited covariance with the cumulative IV opioid dose. The multivariable model was therefore unable to establish an association with cumulative IV benzodiazepine dose and withdrawal. The association between IV benzodiazepine weaning and withdrawal may have been similarly obscured by covariance. Lastly, it is not possible to distinguish the true “reason for intubation” from electronic medical record data, and the study population may exhibit disease heterogeneity.

## CONCLUSIONS

In our single-center study, patients with a higher cumulative IV opioid dose, patients who weaned their IV opioid in the preceding 8–12 hours, and complex care patients had the highest odds of withdrawal. With all other factors held constant, a 0.4 mg/kg decrease in the 4-hour IV opioid dose is associated with 30% increased odds of developing withdrawal 8–12 hours later. Lastly, children who received morphine as their first IV opioid had increased odds of developing withdrawal when compared with fentanyl and hydromorphone, but these results may reflect fentanyl’s prolonged context-sensitive half-life. Further work is necessary to identify at-risk patients and refine preventive measures.

## Supplementary Material
