# Supplementary material for: The Association of Iatrogenic Withdrawal With Opioid and Benzodiazepine Weaning in Children With Bronchiolitis: A Single-Center, Retrospective Cohort Study, 2012–2022
Source: Crit Care Explor. 2026 Mar 13;8(3):e1391. doi: 10.1097/CCE.0000000000001391 (PMC12991735; doi:10.1097/CCE.0000000000001391)
Supplement: Supplementary file 1 [file cc9-8-e1391-s001.pdf]

Appendix A: Supplemental Methods

Age on admission, admission location, ICD codes for bronchiolitis, invasive mechanical ventilation, and the presence of WAT-1 scores during the IV opioid infusion wean were used to identify patients. Age on admission was calculated from birth date and admission date. Patient room numbers were used to determine the admission location. The ICD-9 and ICD-10 codes used to identify bronchiolitis are shown in eTable 1.[1–4] Invasive mechanical ventilation was identified with end-tidal CO_2 documentation; each intubation and extubation date was then confirmed with manual chart review.

Patients were excluded if they had evidence of renal dysfunction (estimated creatinine clearance < 30), tracheostomy, ECMO cannulation, or death during the hospital admission. Patient height and highest creatinine value were obtained for each patient to calculate creatinine clearance. When a height was not documented, it was estimated using the 50th percentile height for the patient weight.[5] The presence of a tracheostomy was determined by ICD-9 and ICD-10 codes, and ECMO cannulation was determined by nurse documentation of ‘clinical events’ (eTable 1).

Other patient data included preferred language, hospital and ICU length of stay, duration of mechanical ventilation, comorbidities, discharge location, and discharge medications.[6] Preferred language was determined via manual chart review of interpreter documentation. Hospital length of stay was calculated from admission and discharge dates. ICU length of stay was calculated from the first ICU transfer to the last ICU discharge. Duration of invasive mechanical ventilation was calculated from intubation extubation dates. Comorbidities included trisomy 21, prematurity, cardiac disease, and the presence of two or more CCC V2 categories.[7] Trisomy 21, prematurity, and cardiac disease were identified by diagnosis codes (eTable 1). Diagnosis codes for trisomy 21 and prematurity were identified by manual review of all diagnosis codes in the cohort. Diagnosis codes for cardiac disease were obtained from CCC V2 (eTable 1).[7,8] Discharge location and medications were determined by manual chart review.

eTable 1: ICD codes, diagnosis codes, and clinical events used to define key study categories

Diagnosis	Classifier Type	Classifiers
Bronchiolitis	ICD codes (diagnosis code: description,)	466.1: Acute Bronchiolitis, 466.19: Acute bronchiolitis, 466.19: Acute bronchiolitis due to human metapneumovirus, 466.19: Acute Bronchiolitis Due to Other Infectious Organisms, 466.19: Bronchiolitis, 481: Pneumococcal Pneumonia [streptococcus Pneumoniae Pneumonia], J21: Acute bronchiolitis, J21.1: Acute bronchiolitis due to human metapneumovirus, J21.8: Acute bronchiolitis due to other specified organisms
Tracheostomy	ICD codes (diagnosis code: description,)	Z43.0: Encounter for attention to tracheostomy, J95.01: Hemorrhage from tracheostomy stoma, J95.02: Infection of tracheostomy stoma, 302323001: Tracheostomy granulation, Z93.0: Tracheostomy status, J95.00: Unspecified tracheostomy complication
ECMO Cannulation	Clinical events (event,)	ECMO Blender, ECMO Blender FiO2, ECMO Catheter Activity, ECMO Catheter Catheter Care, ECMO Catheter Catheter Condition, ECMO Catheter Drainage Description, ECMO Catheter Dressing, ECMO Catheter Flow/Patency Port, ECMO Catheter Infusing, ECMO Catheter Site, ECMO Catheter Type, ECMO CO2 Sweep, ECMO Comments, ECMO Flow cc/min, ECMO Flowmeter, ECMO Heater, ECMO Note,

		ECMO O2 Sweep, ECMO Progress Note text, ECMO Safety Check, ECMO Start Date/Time, ECMO Today/Now, Form-ECMO Progress Note, Hours on ECMO
Trisomy 21	ICD codes (diagnosis code: description,)	Q90.9: Down syndrome, unspecified, Q90.1: Trisomy 21, mosaicism (mitotic nondisjunction), Q91.3: Trisomy 18, unspecified, 68470016: Complete trisomy 21 syndrome, 758: Down's Syndrome, Q90.0: Trisomy 21, nonmosaicism (meiotic nondisjunction), Q90.2: Trisomy 21, translocation
Prematurity	All diagnosis codes (numeric code: description,)	P07.36: Preterm newborn, gestational age 33 completed weeks, P07.30: Preterm newborn, unspecified weeks of gestation, P07.39: Preterm newborn, gestational age 36 completed weeks, P07.22: Extreme immaturity of newborn, gestational age 23 completed weeks, P07.32: Preterm newborn, gestational age 29 completed weeks, 500769016: Chronic lung disease of prematurity, 765.1: Other Preterm Infants, Unspecified [weight], 73793013: Prematurity, 412834018: VLBW - Very low birth weight infant, P07.03: Extremely low birth weight newborn, 750-999 grams, 412710015: Chronic pulmonary insufficiency of prematurity, 454273012: Premature infant 28-37 weeks, 2535575018: Retinopathy of prematurity, 412727011: Apnea of prematurity, 412837013: ELBW - Extremely low birth weight infant, 412889015: Intraventricular hemorrhage of prematurity, 78510010: Anemia of prematurity, 362.24: Retinopathy of Prematurity, Stage 2, 362.2: Retinopathy of Prematurity, Unspecified, V12.49: History of periventricular leukomalacia associated with prematurity, V13.7: Personal history of prematurity, P07.24: Extreme immaturity of newborn, gestational age 25 completed weeks, 617251000124112: Baby premature 35-36 weeks, 2470694018: Retinopathy of prematurity stage 2 - intraretinal ridge, 2470695017: Retinopathy of prematurity stage 3 - ridge with extraretinal fibrovascular proliferation, P07.02: Extremely low birth weight newborn, 500-749 grams, P07.20: Extreme immaturity of newborn, unspecified weeks of gestation, P07.26: Extreme immaturity of newborn, gestational age 27 completed weeks, 362.23: Retinopathy of Prematurity, Stage 1, 362.22: Retinopathy of Prematurity, Stage 0, H35.139: Retinopathy of prematurity, stage 2, unspecified eye, P07.25: Extreme immaturity of newborn, gestational age 26 completed weeks, P07.14: Other low birth weight newborn, 1000-1249 grams, H35.109: Retinopathy of prematurity, unspecified, unspecified eye, P07.37: Preterm newborn, gestational age 34 completed weeks, H35.129: Retinopathy of prematurity, stage 1, unspecified eye, P07.17: Other low birth weight newborn, 1750-1999 grams, P07.35: Preterm newborn, gestational age 32 completed weeks, P07.15: Other low birth weight newborn, 1250-1499 grams, P61.2: Anemia of prematurity, H35.119: Retinopathy of prematurity, stage 0, unspecified eye, P07.16: Other low birth weight newborn, 1500-1749 grams, H35.103: Retinopathy of prematurity, unspecified, bilateral, H35.142: Retinopathy of prematurity, stage 3, left eye, H35.113: Retinopathy of prematurity, stage 0, bilateral, P07.31: Preterm newborn, gestational age 28 completed weeks, H35.131: Retinopathy of prematurity, stage 2, right eye, H35.133: Retinopathy of prematurity, stage 2, bilateral, H35.123: Retinopathy of prematurity, stage 1, bilateral, P07.23: Extreme immaturity of newborn, gestational age 24 completed

		weeks, P07.33: Preterm newborn, gestational age 30 completed weeks, H35.132: Retinopathy of prematurity, stage 2, left eye<br>P07.01: Extremely low birth weight newborn, less than 500 grams, P07.38: Preterm newborn, gestational age 35 completed weeks, P07.18: Other low birth weight newborn, 2000-2499 grams
Cardiac disease[7]	ICD-9 diagnosis and procedure codes (numeric code,)<br><br><br><br><br><br><br><br><br><br>ICD-10 diagnosis and procedure codes (numeric code,)	745.0-745.3, 745.60-745.69, 746, 747.1-747.49, 747.81, 747.89, 35.8, 35.81, 35.82, 35.83, 35.84, 424.0, 424.2, 424.3, 425.0-425.4, 425.8, 429.1, 426.0-427.4, 427.6-427.9, 416.1, 416.8, 416.9, 428.0, 429.3, 428.83, 433.11, V45.81, 996.0, 996.1, 996.61, 996.62, V43.3, V45.0, V53.31, V53.32, V53.39, 00.50, 00.51, 00.53, 00.54, 00.55, 00.57, 17.51, 17.52, 37.41, 37.52, 37.53, 37.54, 37.55, 37.6, 37.60, 37.61, 37.63, 37.65, 37.66, 37.67, 37.68, 37.7, 37.71, 37.72, 37.74, 37.76, 37.79, 37.8, 37.80, 37.81, 37.82, 37.83, 37.85, 37.86, 37.87, 37.89, 37.94, 37.95, 37.96, 37.97, 37.98, 39.81, 39.82, 39.83, 39.84, 39.85, 89.46, 89.47, 89.48, 89.49, 996.83,V42.1, V42.2, V43.2, 37.5, 37.51<br>Q20, Q21.2-Q24, Q25.1-Q26, Q28.2, Q28.3, Q28.9, 02170ZP, 02170ZQ, 02170ZR, 02BK0ZZ, 02LR0ZT, 02LS0ZZ, 02LT0ZZ, 02NH0ZZ, 02RK0JZ, 02RL0JZ, 02RM0JZ, 02RP0JZ, 02RQ07Z, 02RQ0JZ, 02RR07Z, 02RR0JZ, 02SP0ZZ, 02SW0ZZ, 02U70JZ, 02UA0JZ, 02UA3JZ, 02UA4JZ, 02VR0ZT, 02WA0JZ, I34.0, I34.8, I36.0, I36.8, I37.0, I37.8, I42, I43, I51.5, I44, I45, I47, I48, I49.0, I49.1-I49.5, I49.8, I49.9, R00.1, I27.0, I27.1, I27.2, I27.81, I27.89, I27.9, I50.9, I51.7, I51.81, I63.139, I63.239, Z95.1, T82.519A, T82.529A, T82.539A, T82.599A, T82.110A, T82.111A, T82.120A, T82.121A, T82.190A, T82.191A, T82.01XA, T82.02XA, T82.03XA, T82.09XA, T82.211A, T82.212A, T82.213A, T82.218A, T82.221A, T82.222A, T82.223A, T82.228A, T82.518A, T82.528A, T82.538A, T82.598A, T82.6XXA, T82.7XXA, Z95.0, Z95.2, Z95.3, Z95.810-Z95.812, Z95.818, Z45.010, Z45.018, Z45.02, Z45.09, Z95.9, 02H40JZ, 02H40KZ, 02H43JZ, 02H44JZ, 02H44KZ, 02H60JZ, 02H60KZ, 02H63JZ, 02H63KZ, 02H63MZ, 02H64JZ, 02H64KZ, 02H70KZ, 02H73JZ, 02H73KZ, 02H73MZ, 02H74KZ, 02HA0QZ, 02HA0RS, 02HA0RZ, 02HA3QZ, 02HA3RS, 02HA4QZ, 02HA4RS, 02HK0JZ, 02HK0KZ, 02HK3JZ, 02HK3KZ, 02HK3MZ, 02HK4JZ, 02HK4KZ, 02HL0JZ, 02HL0KZ, 02HL0MZ, 02HL3JZ, 02HL3KZ, 02HL3MZ, 02HL4JZ, 02HL4KZ, 02HL4MZ, 02HN0JZ, 02HN0KZ, 02HN0MZ, 02HN3JZ, 02HN3KZ, 02HN3MZ, 02HN4JZ, 02HN4KZ, 02HN4MZ, 02WA0QZ, 02WA0RZ, 02WA3QZ, 02WA3RZ, 02WA4QZ, 02WA4RZ, 03HK0MZ, 03HK3MZ, 03HK4MZ, 03HL0MZ, 03HL3MZ, 03HL4MZ, 03WY0MZ, 03WY3MZ, 03WY4MZ, 0JH600Z, 0JH605Z, 0JH606Z, 0JH607Z, 0JH608Z, 0JH609Z, 0JH60AZ, 0JH60MZ, 0JH60PZ, 0JH630Z, 0JH635Z, 0JH636Z, 0JH637Z, 0JH638Z, 0JH639Z, 0JH63AZ, 0JH63MZ, 0JH63PZ, 0JH70MZ, 0JH73MZ, 0JH800Z, 0JH805Z, 0JH806Z, 0JH807Z, 0JH808Z, 0JH809Z, 0JH80AZ, 0JH80MZ, 0JH80PZ, 0JH830Z, 0JH835Z, 0JH836Z, 0JH837Z, 0JH838Z, 0JH839Z, 0JH83AZ, 0JH83MZ, 0JH83PZ, 0JWT0MZ, 0JWT0PZ, 0JWT3MZ, 0JWT3PZ, 0JWTXMZ, 4B02XSZ, 4B02XTZ, 5A02110, 5A02116, 5A0211D, 5A02210, 5A02216, 5A0221D, T86.20-T86.22, Z94.1, 02YA0Z0, 02YA0Z1, 02YA0Z2

eFigure 1: Recent IV Opioid and Benzodiazepine Medication Doses

Each epoch represents a 4-hour period prior to the WAT-1 score. The total IV opioid and IV benzodiazepine doses administered during each epoch were quantified. All possible differences for each medication class between epochs were then evaluated.

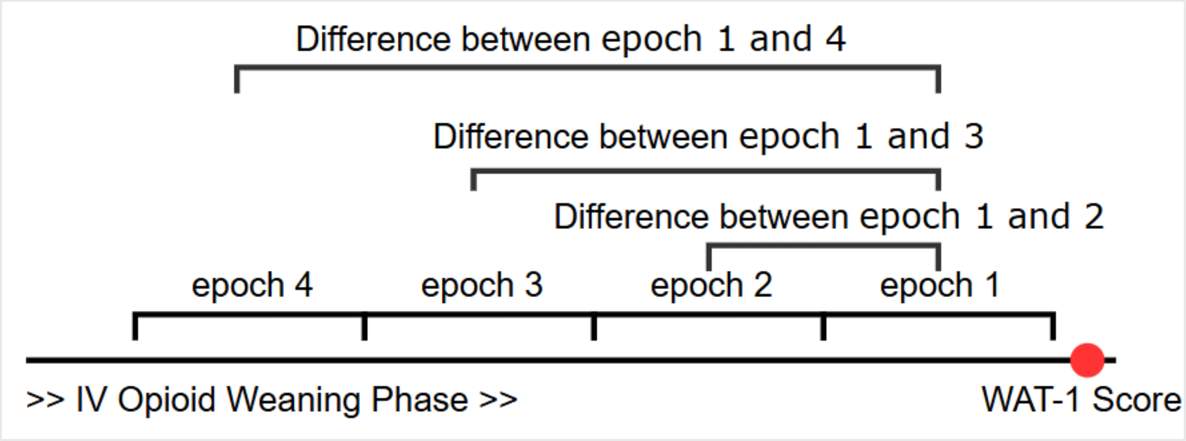

Appendix B: Model Fit Descriptors

Model discrimination was assessed with a) the area under the receiver operating characteristic curve (AUC-ROC), and of the precision-recall curve (AU-PR), b) specificity, precision, negative predictive value, accuracy, and F1 score at sensitivities of 0.8 and 0.9, shown in eTable 2.

AUC-ROC is 00.8974 (95% CI 0.8691, 0.8913), AUC-PR is 0.5888 (95% CI 0.5669, 0.6269), shown in eFigure 2.

Model calibration was evaluated with a) the Hosmer-Lemeshow test (HLt), b) a calibration plot using the observed and predicted proportion of WAT-1 scores indicating withdrawal (eFigure 2), and c) the percentage absolute error of the risk in the calibration plot. Overall association of the variables with the risk of withdrawal was evaluated using the Likelihood ratio test (LRt), compared with a model without predictors. 10,000 bootstrap repetitions were performed for each model parameter estimate, fit performance metric, and confidence interval computation.

The calibration plot demonstrates that the model adequately describes the risk of withdrawal. It is summarized with a regression line using intercept -0.018 (95% CI -0.016, -0.013), slope 1.116 (95% CI 1.088, 1.103), R^2 0.977, (95% CI 0.962, 0.981), percentage absolute error of 0.987 (95% CI 0.856,1.007), and an HLt $p > 0.05$. This indicates a lack of statistical evidence that the predicted risk of withdrawal differs from the empiric risk of withdrawal. The LR test p-value of < 0.01 indicates an overall association between all predictors in the model and the risk of withdrawal.

eTable 2: Model fit descriptors

**Pre-Specified Sensitivity**, 95% CI	0.8001 (0.8, 0.8009)	0.9002 (0.9000, 0.9009)
**Model Fit Descriptor**, 95% CI		
Specificity	0.7877 (0.7778, 0.8165)	0.6799 (0.6656, 0.7179)
Precision	0.4332 (0.4242, 0.4738)	0.3625 (0.3552, 0.3977)
Negative Predictive Value	0.9501 (0.9496, 0.9535)	0.9703 (0.9702, 0.9729)
Accuracy	0.7898 (0.7816, 0.8137)	0.7173 (0.7055, 0.7488)

eFigure 2: Receiver operating characteristic curve of multivariable mixed-effects model

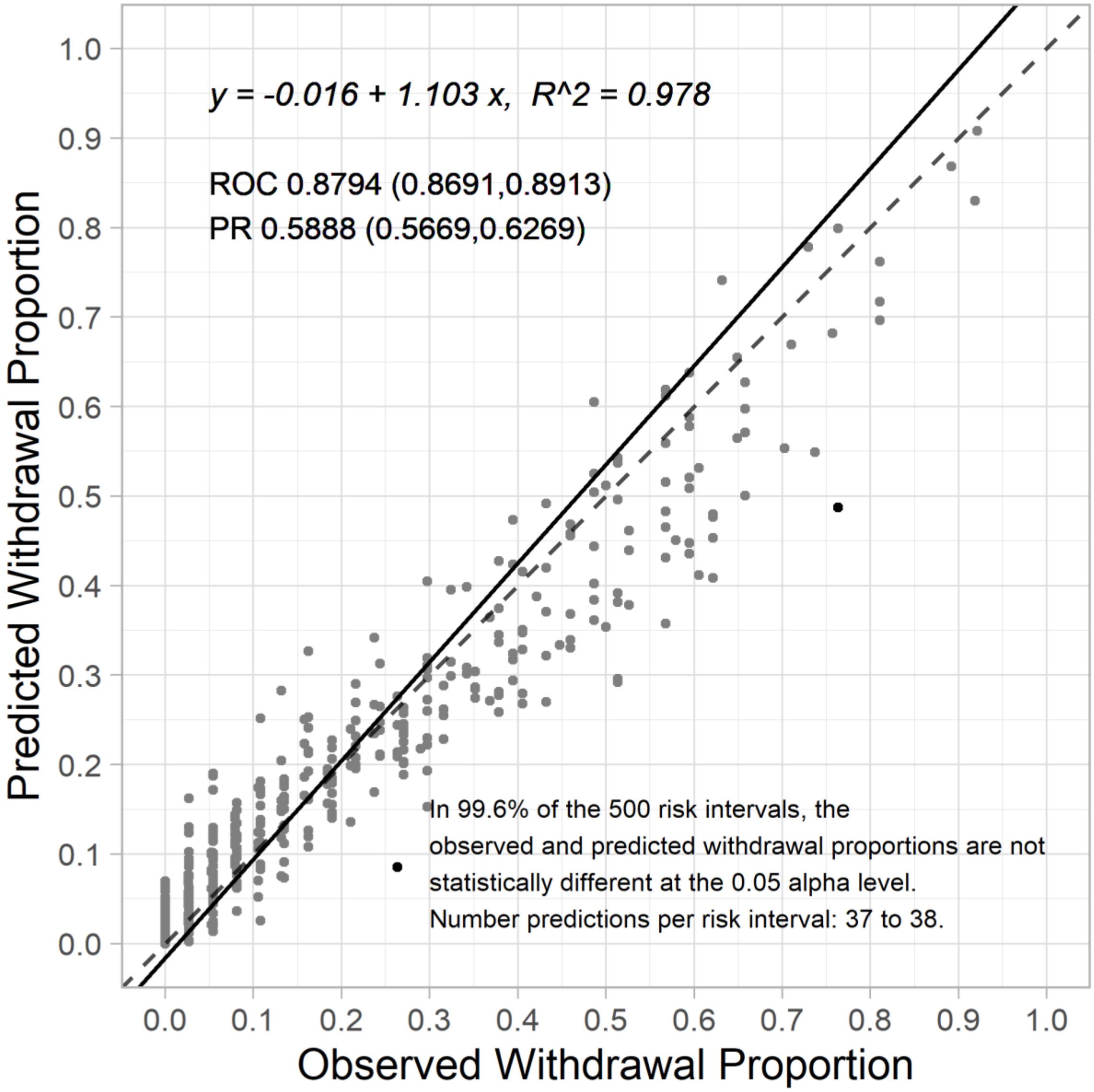

References

1. Shanahan KH, Monuteaux MC, Nagler J, Bachur RG. Noninvasive Ventilation and Outcomes in Bronchiolitis*. *Crit Care Med*. 2021;49(12):e1234-e1240. doi:10.1097/CCM.0000000000005210

2. Wolf ER, Richards A, Lavallee M, et al. Patient, Provider, and Health Care System Characteristics Associated With Overuse in Bronchiolitis. *Pediatrics*. 2021;148(4). doi:10.1542/peds.2021-051345

3. Akenroye AT, Baskin MN, Samnaliev M, Stack AM. Impact of a Bronchiolitis Guideline on ED Resource Use and Cost: A Segmented Time-Series Analysis. *Pediatrics*. 2014;133(1):e227-e234. doi:10.1542/peds.2013-1991

4. Parikh K, Hall M, Teach SJ. Bronchiolitis Management Before and After the AAP Guidelines. *Pediatrics*. 2014;133(1):e1-e7. doi:10.1542/peds.2013-2005

5. Growth Charts. Accessed February 1, 2025. https://www.cdc.gov/growthcharts/index.htm

6. Zurca AD, Suttle ML, October TW. An Antiracism Approach to Conducting, Reporting, and Evaluating Pediatric Critical Care Research. *Pediatric Critical Care Medicine*. 2022;23(2):129-132. doi:10.1097/PCC.0000000000002869

7. Feudtner C, Feinstein JA, Zhong W, Hall M, Dai D. Pediatric complex chronic conditions classification system version 2: updated for ICD-10 and complex medical technology dependence and transplantation. *BMC Pediatr*. 2014;14:199. doi:10.1186/1471-2431-14-199

8. Feinstein JA, Russell S, DeWitt PE, Feudtner C, Dai D, Bennett TD. R Package for Pediatric Complex Chronic Condition Classification. *JAMA Pediatr*. 2018;172(6):596. doi:10.1001/jamapediatrics.2018.0256
